# Awareness challenges of mental health disorder and dementia facing stigmatisation and discrimination: a systematic literature review from Sub-Sahara Africa

**DOI:** 10.7189/jogh.09.020419

**Published:** 2019-12

**Authors:** Susanne Spittel, André Maier, Elke Kraus

**Affiliations:** 1 Charité – Universitätsmedizin Berlin, corporate member of Freie Universität Berlin, Humboldt-Universität zu Berlin, and Berlin Institute of Health, Department of Neurology, Berlin, Germany; 2 University of Bremen, Health Science Bremen – Institute for Public Health and Nursing Research (IPP), Bremen, Germany; 3 Alice Salomon University of Applied Sciences Berlin, Berlin, Germany

## Abstract

**Background:**

Mental health disorders (MHD) are leading causes of disabilities. Awareness of MHD in Sub-Saharan Africa (SSA) is crucial to both health care professionals and general community if those affected by MHD are to be allowed to live in dignity and be socially included, rather than being treated as outcasts or witches, as is presently the case. Therefore, this review aims to map and summarise the extent to which awareness of MHD and dementia in SSA challenges stigmatisation issues.

**Methods:**

A systematic review was conducted using electronic databases (PubMed, CINAHL, PsycINFO). A content analysis of selected studies was performed. Findings on awareness challenges and stigmatisation were identified and categorised.

**Results:**

A total of 230 publications were screened, 25 were selected for this review. The results demonstrate strong supernatural beliefs influencing peoples’ perceptions of diseases. These perceptions promote stigmatising attitudes towards people with MHD and dementia. The education level correlated with stigmatising attitudes, whereby higher educated people were less likely to distance themselves socially from people with MHD and from people living with dementia (PwD). Astonishingly, even people educated in health issues (eg, nurses, medical practitioners) tended to have strong beliefs in supernatural causations of diseases, like witchcraft, and hold negative attitudes towards MHD and PwD.

**Conclusions:**

This review provides some evidence on the influence of traditional beliefs on MHDs in SSA. Those beliefs are powerful and exist in all segments in SSA-communities, promoting superstitious perceptions on diseases and stigmatisation. Awareness and education campaigns on MHD are absolutely mandatory to reduce stigmatisation.

Mental health disorders (MHD) are among the leading causes of disability and are more prevalent among the younger population (0-59 years), whereas chronic diseases such as dementias are more prevalent in older populations (60+ years) [1]. About 1 in 10 persons live with MHD (PwMHD), whilst the provision of mental health care is lacking [2]. According to the World Bank (2016), 84% of the world**’**s population lives in low and middle income countries (LAMIC), 14% in Sub-Saharan African countries (SSA). In many LAMIC, particular in Africa, policies and legislation rarely exist to establish essential mental health services [3,4]. Data from Africa show highest scores in illiteracy and lowest scores in expenditure on mental health, mental health resources, and lowest disability-adjusted life years by neuropsychiatric conditions [3]. Besides, SSA counts a high prevalence of infectious diseases, which impacts the prevalence of probable common MHD (eg, HIV-related dementia) [5-8]. For instance, estimated HIV-related dementia incidence cases in studied populations with HIV/AIDS varies up to 36% [9]. Moreover, the number of people living with dementia (PwD) is increasing – especially in LAMIC as numbers rise more prominently compared to the developed world [10,11]. By 2050 the number of PwD will more than triple in LAMIC [11], caused by infectious diseases like tuberculosis or HIV, but also by a growing middle class population of Africa with an improved sustainable livelihood that allows them to seek better health care, which in turn is likely to contribute to life expectancy, morbidity and mortality rates [8,12]. However, most of these conditions are insufficiently diagnosed and remain untreated [13,14]. Furthermore, PwMHD often experience stigmatisation; even their families, and friends as well as health care providers experience the impact of public stigma [15]. Perceiving stigma is strongly associated with MHD and occurs more frequently in developing countries [16]. Worldwide, people from various cultures hold different traditional beliefs, which can be powerful in healing people on the one hand, but on the other hand, strong beliefs can also affect attitudes towards PwMHD, even amongst people that have received medical education [17]. Supernatural attitudes to an illness exist especially in societies where traditions dominate society [18]. In most SSA-cultures, people strongly believe in supernatural powers [19,20]. As long as there is no awareness and “name” for diseases (eg, for dementia), people’s behaviour – especially if people are seen as acting “strange” – can be misinterpreted and lead to accusation and exclusion [21].

## Objective

This review aims to identify awareness and knowledge as well as attitudes (eg, beliefs, feelings, and behaviours) around MHD and dementia in SSA that reveal stigmatisation. Hypothetically, it is assumed that supernatural traditional beliefs challenge perceptions and concepts on MHD and dementia and result in stigmatisation of people living with such diseases in SSA. The review targets peoples’ awareness and knowledge of MHD and dementia, their attitudes towards PwMHD and PwD, as well as the stigmatisation grounded in supernatural beliefs.

## METHODS

### Data sources and search strategy

A systematic literature review was conducted using online databases in accordance with the PRISMA statement [22]. Different search strategies were performed systematically to identify published studies reporting on awareness and knowledge around MHD and dementia in SSA that reveals stigmatisation. The following electronic databases were used: PubMed^®^ including MEDLINE^®^, CINAHL and PsycINFO^®^. Key words included ‘mental health’, ‘dementia’ or ‘Alzheimer*’, and ‘education’ or ‘awareness’, and ‘stigma*’ or ‘witch*’, and ‘Africa’. Within the databases, key words were searched in combination. Title and abstract of articles were screened for relevance based on inclusion criteria. Reference lists were hand checked to identify supplementary studies, and a “grey” literature search was conducted. Additionally, articles, books, chapters, reports, non-empirical studies, and commentaries were used for a more in-depth understanding.

### Study selection

For review inclusion, studies based on following criteria were selected. Studies were required to (a) focus on MHD or dementia, especially focussing on diseases which can cause abnormal “strange” behaviour in the affected persons; (b) address awareness or education, and (c) report on stigmatisation on those diseases. Because of differences on cultural values and beliefs between Northern Africa (Arabic culture) and SSA (Gupta, Hanges & Dorfman, 2002), articles were required (d) to relate to SSA. Studies published in English or German (e) were included. There was no time limitation for inclusion.

### Outcomes of interest

Primary outcome of interest was an influencing factor on the awareness and concepts on MHD and dementia. Traditional beliefs in Sub-Sahara Africa such as witchcraft were considered as influencing factors. Additionally, stigmatisation issues concerning MHD and dementia and its grounding in supernatural beliefs were of interest.

### Data extraction and quality assessment

In a first step data were extracted by country where results were conducted, method of the study, participants and sample size, and the objective of the study. In a second step data were extracted reporting on concepts on MHD and dementia, attitudes towards, and stigmatisation of people living with MHD and dementia. Extracted data were classified separately for health care professionals (HCP; eg, nurses, caregivers, medical practitioners, care-providers), and general community members (GCM; eg, community members, children, and clergy). Full texts of selected studies were checked for representation of sampling, quality of methods applied, and reporting strategy.

## Results

### Systematic review

**Figure 1:**
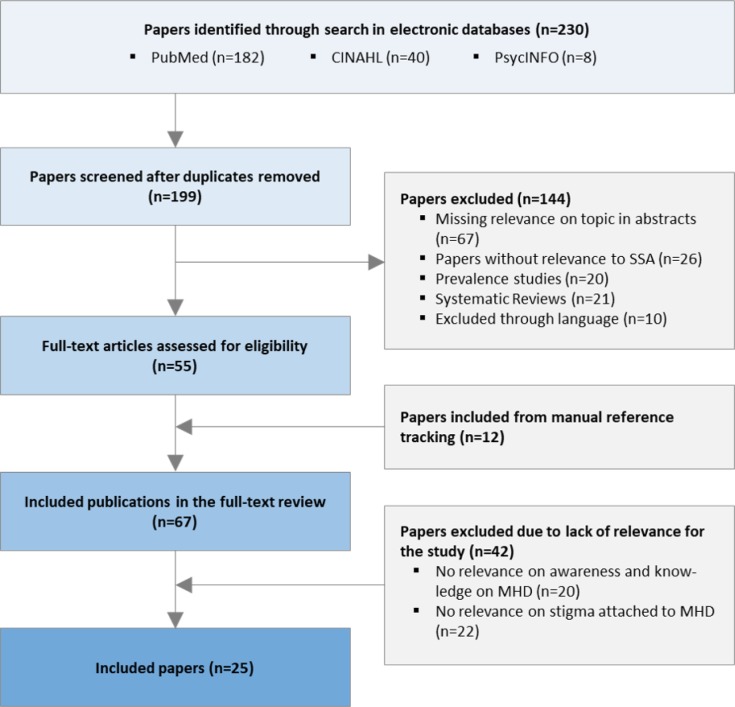
Flow-chart of search strategy used to identify the studies relevant for the review. MHD – mental health disorders, SSA – Sub-Saharan Africa.

In total 230 potentially relevant publications were identified via systematic search in the electronic databases: PubMed (182 references), PsycINFO (8 references), CINAHL (40 references). After removing duplications, papers were screened based on title and abstract. 55 relevant publications were selected for further examination. In the final selection, 25 articles met all relevant inclusion criteria and were involved (**Figure 1**).

### Study characteristics

Most studies focussed on awareness challenges on MHD in general (n = 21), four studies investigated dementia. Reviewed studies originated from *Nigeria* (n = 10), *Ghana* (n = 2), *Republic of Congo* (n = 1), *Uganda* (n = 2), *Ethiopia* (n = 2), *Tanzania* (n = 1), *Zambia* (n = 1), *Zimbabwe* (n = 1), *South Sudan* (n = 1), and *South Africa* (n = 4).

Methodologies included studies applying a quantitative approach by retrieving questionnaires (n = 13) or a qualitative design by conducting interviews or focus group discussions (n = 10). Two studies combined quantitative and qualitative methods. Main results were categorised according to the following themes: (I) concepts, and knowledge on MHD and dementia, (II) perceptions on witchcraft, (III) attitudes towards PwMHD and PwD, and (VI) stigmatisation of PwMHD and PwD. Characteristics of the reviewed studies are summarised in **Table 1**.

**Table 1:** Characteristics of included studies

No.	Author, year	Country	Method	Participants, sample size	Aim
1	Adebiyi et al., 2016 [23]	Nigeria	FGD	GCM, n = 6	Exploration of attitudes towards PwD.
			Questionnaire-survey	GCM, n = 313	
2	Adewuya, Oguntade, 2007 [19]	Nigeria	Questionnaire-survey	Medical doctors, n = 312	Evaluation of attitudes towards PwMHD.
3	Adewuya, Makanjoula, 2008a [24]	Nigeria	Questionnaire-survey	GCM, n = 2078	Evaluation of beliefs causing MHD.
4	Adewuya, Makanjoula, 2008b [25]	Nigeria	Questionnaire-survey	GCM, n = 2078	Assessing attitudes towards PwMHD.
5	Audu et al., 2013 [26]	Nigeria	Questionnaire-survey	GCM, n = 325	Examination of stigmatisation of PwMHD.
6	Ayazi et al., 2014 [27]	South Sudan	Cross-sectional-survey	GCM, n = 1200	Investigation of attitudes towards PwMHD.
7	Barke et al., 2011 [28]	Ghana	Questionnaire-survey	GCM, n = 508	Measuring attitudes towards PwMHD.
8	Dogra et al., 2012 [29]	Nigeria	Cross-sectional-survey	Schoolchildren, n = 164	Assessing views and knowledge about MHD.
9	Egbe et al., 2014 [30]	South Africa	Interviews, FGD	HCP, n = 32, Healthcare users, n = 45	Exploration of psychiatric stigma in order to inform interventions.
10	Faure-Delage et al., 2012 [31]	Congo	Questionnaire-survey	PwD, PwMHD, n = 117, Relatives, n = 123	Exploring people’s perception about dementia.
			Interviews	HCP, n = 33	
11	Girma et al., 2013 [32]	Ethiopia	Cross-sectional-survey	GCM, n = 845	Measurement of public stigma against PwMHD.
12	Girma et al., 2014 [33]	Ethiopia	Cross-sectional-survey	GCM, n = 845	Investigation of the degree of family stigma.
13	Gureje et al., 2005 [34]	Nigeria	Questionnaire-survey	GCM, n = 2040	Determination of knowledge and attitudes of MHD.
14	Gureje et al., 2006 [35]	Nigeria	Questionnaire-survey	GCM, n = 2040	Examination of causation and attitudes.
15	Igbinomwanhia et al., 2013 [36]	Nigeria	Cross-sectional-survey	Clergy, n = 107	Determination of attitudes towards PwMHD.
16	Kabir et al., 2004 [37]	Nigeria	Questionnaire-survey	GCM, n = 250	Examination of knowledge and attitudes of MHD.
17	Kapungwe et al., 2010 [6]	Zambia	Interviews, FGD	Stakeholders, n = 56	Exploration of causes of MI to address stigmatisation against PwMHD.
18	Mavundla et al., 2009 [20]	South Africa	Interviews	Informal caregivers, n = 8	Exploration of experiences of informal family caregivers of PwMHD.
19	Mkhonto, Hanssen, 2017 [38]	South Africa	Interviews	Relatives, nurses, n = 8	Exploration of the link between culture and dementia care.
20	Mohamed-Kaloo, Laher, 2014 [39]	South Africa	Interviews	General practitioners, n = 10	Investigation of perceptions on MHD.
21	Mushi et al., 2014 [40]	Tanzania	Interviews	PWD n = 25, PWDs’ caregivers, n = 16	Exploration of socio-cultural beliefs on dementia.
22	Ofori-Atta et al., 2010 [41]	Ghana	Interviews, FGD	Stakeholders, n = 88	Exploration of causes of MHD.
23	Patel et al., 1995 [42]	Zimbabwe	FGD	HCP, n = 76	Generation of concepts of MHD.
24	Quinn, Knifton, 2014 [43]	Uganda	Interviews, FGD	Stakeholders, n = 18	Understanding beliefs and stigma associated with MHD.
25	Ssebunnya et al., 2011 [44]	Uganda	Interviews, FGD	Stakeholders, n = 68	Exploration of perceptions on the mental health care system.

FGD – focus group discussion, MHD – mental health disorders, PwMHD – people living with mental health disorders, PwD – people living with dementia

### Concepts and knowledge on MHD and dementia

The review reveals that people in SSA hold different explanations on the causation of MHD and dementia. Explanations derive from biological, psychological, and spiritual concepts, or other explanations, classified and summarised in **Table 2**.

**Table 2:** Classification of concepts on MHD and dementia

Category	Concept on MHD	GCM in %	HCP in %	Representative studies
Biological explanations	Brain illness*	R: 0.9-38.0*	81.8*	[26,31,34]
	Brain infection	R: 25.0-30.4†	49.4	[19,31]
	Heredity	R: 10.3-38.3	32.7	[19,24,25,29,34]
Psychosocial factors	Stress*	R: 6.7-80.8†	58.3 †	[6,19,24,29,31,32,34,37,39,40,42,43]
	Personal failure	R: 10.2-61.2	20.2 †	[19,24,28,29,36]
	Drug, alcohol misuse	R: 9.5-80.8†	67.9 †	[6,19,24,26,32,33,37,42]
Supernatural explanations	Witchcraft, evil spirit*	R: 19.9-65.5†	53.8 †	[6,19,20,24-27,29-34,37-43]
	Punishment by God*	R: 9.3-50.1†	6.4 †	[6,19,24-26,31,32,34,37,40,43]
Other explanations	Madness*	†	†	[6,23,41-44]
	Normal part of ageing*	R: 18.2-71.9†	†	[23,31,40]
	Contagious disease*	13.2*	†	[6,31,43]
	“Whites peoples disease”*	n/a	†	[38]

MHD – mental health disorders, GCM – general community members, HCP – health care professionals (eg, medical practitioners, nurses, caregivers), R – Range, n/a – not applicable

*Addressed by dementia studies.

†Qualitative results involved.

Biological explanations are used least often to describe MHD’s or dementia’s causation. In terms of MHD, HCP are more likely to use biological explanations compared to GCM [19,24]. Also, PwD themselves or their relatives and caregivers, mostly tended to lack knowledge of dementia [31,40].

Psychosocial explanations are considered to be more prominent causes. Especially misuse of drugs and alcohol are most frequently seen to cause MHD (up to 80%); followed by stress (up to 81%) or the burden of poverty (53%) [24,32,34].

Furthermore, misconceptions exist very frequently in the way that MHD or dementia are not perceived as diseases but as madness, a source of witchcraft or sorcery (up to 66%). Those supernatural explanations are predominantly considered to cause MHD and dementia occurring in all segments of communities, including PwMHD itself and HCPs [34]. Especially people lacking knowledge of the disease aetiology, witchcraft was identified as the cause [20,41]. The behaviour of PwD is perceived as strange, abnormal, and dangerous which makes people think about witchcraft [30,38]. For instance, Zambian interviewees acknowledged: “*This is a mad person [...]”; “People still think that mental illness is caused by evil spirits. […];”* or that PwMHD are bewitched [6]. In Ghana, it is common that people tend to understand MHD as the “work of witches’: *“People don’t really understand the causes, so they attribute it to witches […]”; “People see madness, epilepsy and witchcraft as all the same. They see mental illness as the doing of witches and their curses”* [41]. Likewise, informal caregivers from South Africa, who do not understand the illness of their relatives, believed their disease was caused by the environment or witchcraft [20]. Moreover, high numbers of medical doctors (in Nigeria over 50%) saw spiritual explanations as the aetiology of MHD [19].

### Belief in witchcraft

Spiritual beliefs, like ‘witchcraft’, ‘Satanism’, ‘evil spirits’ or ‘punishment from God’ were found to be very strong and leading to misunderstanding the aetiology of MHD or dementia in the following countries: *Nigeria* [19,24,26,29,34,37], *Ghana* [41], *South Africa* [20,30], *Uganda* [43], *Tanzania* [40], *Zambia* [6], *Zimbabwe* [42], *South Sudan* [27], and *Ethiopia* [32]. Those spiritual beliefs, seen as causing diseases, are also associated with negative attitudes towards PwMHD and higher stigmatisation. They can prevent people from seeking professional help, can impact concepts of caregiving, and lead to marginalisation of PwMHD [20,25,27,30,34,43].

### Attitudes towards PwMHD and PwD

Negative attitudes and stigmatisation against PwMHD are found to be widespread in these countries: *Nigeria* [23], *Ghana* [28,41], *Republic of Congo* [31], *Tanzania* [40], *South Sudan* [27], *Uganda* [43,44], *Ethiopia* [33], *Zambia* [6], *Zimbabwe* [42], and *South Africa* [38]. Negative attitudes are held by different groups in society – most evident among GCM, but also within families, among HCP and health care users, as well as within the government level or among policy makers [6,23,24,26-28,30,33,35,37,41,43]. Also, even if people were friends with PwMHD, they hold negative attitudes towards them, which was found in *South Africa* and *Zambia* [6,30]. The finding that negative attitude towards PwMHD are not limited to adults was established in a Nigerian schoolchildren survey [29].

Generally, PwMHD or PwD are perceived to be dangerous or aggressive, to be a burden to society as well as a public nuisance. Among both GCM and HCP, perceived dangerousness or aggressiveness are two of the main prejudiced perceptions held towards PwMHD, as the majority of reviewed studies indicate (**Table 3**).

**Table 3:** Attitudes and prejudices towards PwMHD and PwD

Attitudes and prejudices*	GCM in %	HCP in %	Representative studies
Are dangerous	R: 57.9-96.7‡	70.5‡	[6,19,25,27,34,36,43,45]
Show a violent/aggressive behaviour	R: 21.8-96.7‡	R: 51.0-53.8‡	[6,19,20,26,27,29,30,32,34,35,37,42]
Are frightening or scary†	R: 23.3-86.4‡	R: 26.9-37.5‡	[6,19,20,26,27,30,32,34-38]
Making others ashamed†	R: 11.3-86.1‡	26.9‡	[19,23,25,27,29,33-35,39,43]
Are a burden to individuals, families, and communities†	R: 37.3-56.3‡	‡	[20,28,31,36,40,43]
Are different; behave strange†	R: 33.4-79.7‡	‡	[28,31,32,36,38]
Are a public nuisance	R: 15.0-97.8	n/a	[26,34,35]
Are unpredictable or lacking self-control	57.9	R: 59.0-85.9	[19,29]

PwMHD – people living with mental health disorders, PwD – people living with dementia, GCM – general community members, HCP – health care professionals (eg, medical practitioners, nurses, caregivers), R – range, n/a – not applicable

*Attitudes are listed in order of the relevance mentioned in the papers reviewed.

†Addressed by dementia studies.

‡Results from qualitative studies involved.

Such negative perceptions can arise from fear, focusing on the hardship of caregiving or lacking interest, motivation, and knowledge around MHD [19,20,44]. A South African study disclosed that GCM and family members are afraid of the PwD and that even nurses hold such attitudes until they started working with them [38]. This not only leads to misinterpretation of behaviours shown by PwMHD or PwD but also often relates to higher levels of stigmatising attitudes [6,25,27,37,43].

Stigmatising attitudes can also concern the family of or people caring for PwMHD, professional health care providers, or whole mental health institutions [6,20,25,30,36,44]. The education level correlates with stigmatising attitudes, whilst people with higher education tend to keep less social distance towards PwMHD, or their relatives [26,28,33]. People with less knowledge show higher social distance [6,20,27,29,35,36,39,44,46]. Those attitudes and perceptions, especially being afraid of having conversations with PwMHD or believing that the presence of PwMHD might pose a risk to unaffected people, is observed to strengthen social isolation [25,28,29].

### Stigmatisation of PwMHD and PwD

In particular, social distance and exclusion of the PwMHD from the community (eg, keeping the ill person behind locked doors) is mainly addressed by the reviewed studies [6,20,27,28,30,32,33,36,39-44]. Both GCM and HCP would feel disturbed about sharing a room or their house with PwMHD or living next door to someone who is mentally ill, or even having PwMHD living in residential neighbourhoods (**Table 4**). A Zimbabwean study revealed that PwD are locked away as long as family members are reluctant to disclose the status of their relatives [40]. Even within families, relatives exclude their ill family members, for example, as these have to eat separately or are forced to live on the street [43]. It is frequently found that PwMHD are unable to lead normal lives because of social isolation [30,42]. Zambian stakeholders disclosed that PwMHD live in misery and loneliness without hope while community members bully and throw stones at them [6]. A Ghanaian study describes that especially women experience widespread physical abuse as communities force persons suspected of using witchcraft to move to designated areas called ‘witch-camps’ where these women undergo ‘cleansing’ rituals to rid them of witchcraft. They may also be expelled by the community, or even chased away by people intending to kill them [41]. In addition, the reviewed studies reveal stigmatisation through maltreatment (eg, being beaten by family members, or tied to a tree), and the point to influences on the health situation of PwMHD (eg, obtain service in a delay or being ignored by health care providers), as well as that PwMHD usually experience job disadvantages (**Table 4**).

**Table 4:** Perceived stigma of PwMHD and PwD

Category	Perceived stigma	GCM in %	HCP in %	Representative studies
Social isolation/distance	Avoid contact /the person	R: 23.3-86.4	n/a	[19,25-29,34,36,37,45]
	Unwillingness of maintaining a friendship	R: 51.4-83.1	n/a	[25,27,29,33,34]
	Not marry*	R: 18.6-96.6†	80.8†	[6,19,23,25-28,33,34,36,43]
	Not share room	R: 47-84.5	64.1	[19,25-27,34,35]
	Locked away / keep behind Locked doors*	R: 24.3-47.8†	†	[28,33,36,39,40,43]
	Isolation from the community*	R: 24.3-42.1†	†	[6,20,28,30,31,36,38,39,41-43]
Physical torture	Throwing stones*	†	n/a	[6,38]
	Chained or tied them up	†	n/a	[30,43]
	Maltreatment or beaten*	†	n/a	[30,38,44]
Mental torture/burden	Living neglected, rejected, or blamed*	65.5†	†	[6,20,23,29,30,33,38,41-44]
	Being laughed at, or bullied*	†	n/a	[23]
	Being shunned*	†	n/a	[23]
Worsened health situation	Ignored by health care staff	†	†	[6,20,30]
	Traditional healers instead of medical care*	R: 18-34†	n/a	[20,37-40,42]
	Inadequate or delayed health	†	†	[6,30,36]
	Mental health service out of residential areas	R: 39.8-69.1	n/a	[28,36]
Job/education burden	Work with a PwMHD	R: 43.1-84.8†	R: 32.1-39.7†	[19,25,27,34,42,44]
	Getting no or filthy jobs	†	†	[6,43]
	Schooling or studying with a PwMHD	70.2†	n/a	[29,43]
	Employers passing applications over	77.2	n/a	[28]

PwMHD – people living with mental health disorders, PwD – people living with dementia, GCM – general community members, HCP – health care professionals (eg, medical practitioners, nurses, caregivers), R – range, n/a – not applicable

*Addressed by dementia studies.

†Qualitative results involved.

Moreover, the stigma can influence whole families, HCP, or whole mental-health care hospitals. Also, families or carers of PwMHD often feel lonely and experience social isolation. Because of their involvement in care, they are often blamed by community members, eg, cannot attend church sessions, funerals, or other important traditional functions [20,33,42]. Even a whole neighbourhood can be stigmatised if PwMHD live there [6]. On the other hand, caregivers have to cope with stress, burnout through physical aggression, and destructive behaviour from PwMHD or PwD [31,40]. Another major challenge in providing care are financial constraints, eg, having problems with maintaining employment [20,42].

### Relationship between findings

The findings suggest that generally attitudes of people with poor knowledge, towards PwMHD were predominantly negative [35]. In addition, it was found that people assigning psychological causes to the medical conditions yield more positive attitudes towards PwMHD [33,35]. In contrast, people holding spiritual views of diseases’ causations show higher social distance towards PwMHD [25,27,30,34,43]. It can be postulated that such negative attitudes may reflect a lack of understanding of clinical subtleties of MHD [36]. For example, an Ethiopian study revealed that people with awareness of diseases’ signs and symptoms have less stereotyped attitudes and prejudices towards PwMHD [33]. Fortunately, people who cared for PwMHD or PwD, or people with knowledge of diseases causation show less stigmatising attitudes as they started to understand the disease [25,38]. Interviewed caregivers of PwD from Tanzania stressed the need for improved knowledge of care and support for PwD because of carer burden [40].

## DISCUSSION

This appears to be the first systematic review based on studies focusing on awareness challenges of MHD and dementia in SSA and the related stigmatisation issue. Based on the results of this review, there is evidence that supernatural beliefs influence awareness and knowledge on MHD and this results in the stigmatisation of people living with those conditions in SSA. The review furthermore highlights findings on people’s attitudes towards PwMHD and PwD, their awareness and knowledge of those conditions, and the issue of stigmatisation, grounded in supernatural beliefs.

### Discussion of essential key findings

The 25 studies reviewed clearly show that people in SSA generally hold strong negative attitudes towards PwD [23,40,47] and PwMHD [6,19,34]. Negative attitudes are widespread in SSA as they occur in Western-Africa, Eastern-Africa, and Southern-Africa [20,30,48]. Those negative attitudes are held by GCM and also by HCP, and arise precisely because people hold misperceptions on MHD or dementia [38]. It became clear that supernatural beliefs (eg, witchcraft, sorcery, evil spirits) play an important role to explain behaviours of PwMHD or PwD living in SSA as they influence people’s concepts on causation of diseases. Such supernatural beliefs are still widespread and strong in SSA, eg, in West- and East-African Countries as well as in countries in Central- and Southern Africa [6,19,33,40-42,49]. Based on these findings it can be concluded that people possessing those traditional beliefs appear to be influenced in their understanding of diseases, regardless of whether they live in SSA or in other parts of the world. On the positive side, higher level of education or experience with caring for people living with such conditions can be seen as indicators for biological models of causation, reducing supernatural explanations for such diseases [20,27,38,41]. Awareness of MHD and dementia could help to lessen stigmatisation and minimise exclusion of PwMHD, PwD, and those who care for them [50]. However, the review also shows that PwD themselves, their caregivers, and HCP relate dementia to curses or witchcraft [20,38,40,42]. Alarmingly, more than half of medical Nigerian practitioners see witchcraft as potentially causing MHD [19]. This high percentage underpins the strong influence of supernatural beliefs on causation of diseases. It could be found that those issues are also relevant for other diseases like epilepsy, what results from Eastern and Central African studies [49,51]. Likewise, future HCP like Ugandan medical students believed in supernatural causes (eg, witchcraft) of epilepsy [49].

As long as HCP misinterpret the aetiology of MHD because of supernatural beliefs, hardship remains for PwMHD or PwD and their families to develop awareness and knowledge on their conditions. To this effect, awareness campaigns and mental health literacy programs are urgently needed [28,30,39,40]. Community awareness campaigns as well medical curricula need to deal with the impact of cultural beliefs [19].

**Figure 2:**
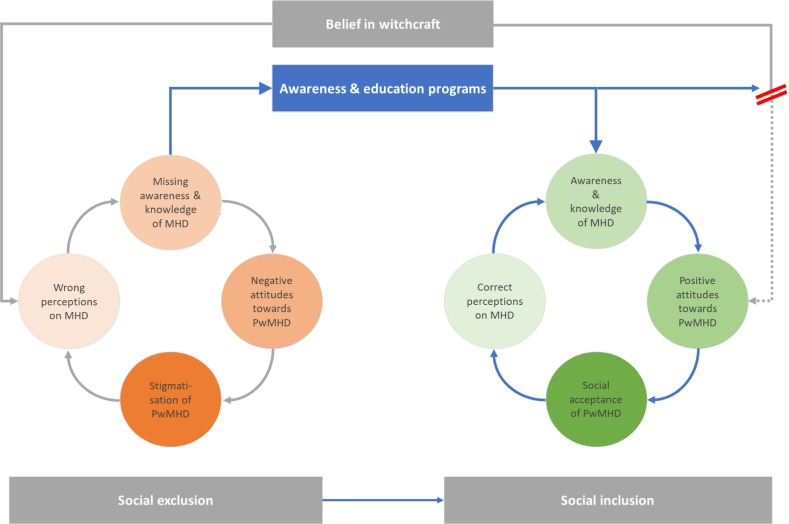
Summarisation of findings in circular flow of awareness challenges in Sub-Saharan Africa. MHD – mental health disorders, PwMHD – people living with mental health disorders.

As seen in **Figure 2**, it can be summarised that without such investigations – particularly in countries where awareness and knowledge on diseases lacks – supernatural beliefs will strengthen misperceptions about MHD and dementia. As long as people hold misperceptions of the causes of diseases, people will be less likely to obtain knowledge about diseases, or even to find the underlying cause of PwMHD’s or PwD’s behaviours. Those beliefs, as well as a lack of awareness and knowledge is likely to promote negative attitudes and stigmatisation. If a person experiences stigmatisation, like physical abuse, exclusion and loneliness [6,41], this can lead to anxiety, fear and stress for that person and can promote a higher risk for dementia [52]. A comparative analysis of anxiety, fear and stress caused by witchcraft and its effects promoting MHD or dementia, was not in the scope of this review. However, studies on this concern would be of interest in SSA. It is generally very important that research be conducted by local researchers that have a deep understanding of the beliefs around witchcraft, since they are more likely to identify relevant factors that influence both the cause and the dealing with dementia.

Without raising awareness of those who wrongly stigmatised or accused PwMHD or PwD, misperceptions and concepts are likely to prevail. Helping PwMHD and PwD to live safely and in dignity, communities and neighbourhoods, health care workers, and politicians need to be educated about disease symptoms to understand the behaviour of people living with such diseases. Greater awareness and understanding people’s behaviours could help change people’s attitudes in a positive way, eg, lessening fear of PwMHD or PwD. Perceived dangerousness has been found as the main negative perception people held towards MHD, and these perceptions are most likely to promote discrimination [36]. Curbing social isolation and understanding ‘strange’ and ‘abnormal’ behaviour against the background of health care science might counter false accusation. Awareness and education could prevent prejudice that base people’s behaviours on supernatural beliefs and instead enhance positive attitudes, as well as promote people’s health status, social acceptance, and social inclusion. It is very important and mandatory for GCM and HCP to understand the aetiology of such conditions and it is fundamental to treat PwMHD or PwD with respect so that they can live in dignity, even if they show a violent and dangerous behaviour. Awareness through education will help to interrupt the circular flow of stigmatisation.

### Limitations

Since there is generally a lack of studies addressing awareness challenges of dementia and related stigmatisation issues in SSA, this review also included studies facing the review’s topic on MHD in general. Although there are similarities in the findings, the degree to which these results can be generalised is limited.

Moreover, the review focused on certain regions in SSA. There is a shortage of studies conducted especially in different Central and Southern African Countries. In this context, studies conducted among African communities located outside SSA have not been included. Furthermore, the review was limited to inclusion of English and German studies. Studies from francophone African countries and published in French might have given further insight into the topic. Even though some studies showed differences of stigmatising attitudes among rural and urban residents, or among different income situations and gender on those differences has not been focussed on [27,29,33,34,37].

## Conclusion

It can be concluded that supernatural beliefs influence misperceptions of causes of dementia or other mental health disorders. Hence, such perceptions also influence people’s concepts on diseases. The misperceptions are likely promoting negative and stigmatising attitudes towards people living with such conditions. Such beliefs are powerful and exist in all segments in Sub-Saharan African communities. Surprisingly, although people with a professional health education background like nurses and medical practitioners also often believe in causes such as witchcraft for diseases. Education campaigns on dementia disease and mental health disorders are mandatory to create greater awareness and knowledge on such conditions and its causes. Due to the results, which show that traditional beliefs influence the knowledge about diseases, they should play a role in the explanation of disease causes. Those influences should be also addressed in medical health curricula. In order to reduce stigmatisation and discrimination caused by the belief in witchcraft or other supernatural powers, researchers need to focus on a wide range of measures including awareness raising, so that the underlying causes for these practices are addressed as well. Furthermore, laws on witchcraft, like the Witchcraft Suppression Acts of 1957 of the Parliament of South Africa, to provide for the suppression of the practice of witchcraft and similar practices (eg, prohibition of various activities related to witchcraft or witch-hunting; South African Law Reform Commission, 2014), should be enacted or reviewed and reformed all over Sub-Saharan Africa. Even though there are legislations to prohibit witchcraft, those legislations need to be known and respected by societies to reduce the fear of witchcraft and all its consequences.
